# Patient experience from the vantage point of a hospitalized patient safety expert: a personal commentary

**DOI:** 10.1186/s13037-021-00307-4

**Published:** 2021-10-03

**Authors:** Narinder Kapur

**Affiliations:** University College London, London, UK

*As Psychology / Human Factors advisor to the UK surgical patient safety learning group (CORESS), and having published on clinical excellence and patient experience issues, the author recently had the unique experience of being a surgical patient. The author gained insights and learned lessons that could help improve the patient hospital experience and also patient safety.*

Most people will end up in hospital for one reason or another, and sadly this experience is now more common as a result of COVID-19. It is vital that not only is high quality care provided but also that the patient experience is a positive one. For the past 8 years, I have been Psychology / Human Factors advisor to CORESS, the Confidential Reporting System for Surgery group (www.coress.org.uk) and publications have emanated from that work [1, 2]. I recently spent 8 days in hospital having a major surgical procedure, lumbar decompression. The procedure went well, and my care was excellent, but I did learn a lot about the patient hospital experience, and this learning could benefit others. Some of my suggestions may also impact on patient safety.

Patients usually come into hospital for an investigation, for a planned procedure or as an emergency. Especially if it is a planned procedure, as well as being told what to bring to hospital they should be given a leaflet about any pre-operative preparation for the procedure, the procedure itself and its consequences in the days/weeks/months afterwards and how best to deal with them, especially if it is a major procedure. Naturally, discussion of the procedure and risks, etc. will usually have been given earlier in an outpatient clinic setting, but having something tangible in the days/weeks before the procedure would help address concerns and psychologically prepare the patient. With the advent of the internet, there are many helpful resources, including YouTube videos relating to surgical and other procedures. Links to a selection of the best of these videos should be included in the information leaflet provided to patients. If there is a patient TV/video screen system in the hospital, such documentation and video material could be made available on the system. There will also have been local patients who have been through the procedure, and contact details of any who have agreed to be spoken to by the patient in question (by phone, video call, in person) about the experience of the procedure would also be helpful to provide. Perhaps they could be called ‘patient buddies’. If patients are given a general welcome pack when they arrive, this should be given in person with the contents explained (mine lay on a table for 5 days before I saw it!). Having hard copies of relevant documents displayed on a ‘Resource Wall’ on the ward could benefit both patients and junior staff.

When patients are on bed-rest after surgery, and if there is no catheter in place, they may use a bed urinal while lying in bed or sitting on the bed-side. When we pass urine, we usually wash our hands afterwards. An antimicrobial handwipe should always be given along with the urinal, so that some form of hand-washing takes place after urination.

During my hospital stay, I unfortunately spilt boiling water over myself and then fell to the ground while trying to make myself a cup of tea. Because I had not yet added milk, and since hospital gowns are paper thin, I had first- and second-degree burns. This just reminded me about the general risks of hot fluids, and apart from general precautions about offering tea/coffee in beakers to vulnerable patients, I wonder whether if tea/coffee is ever offered in a cup without milk, then it should be allowed to cool for a few minutes so that the water is no longer boiling. Also, refuse to entertain requests from people like me who bring their own thermos flask, ask for it to be filled with boiling water, and then try and make their own speciality cup of tea!

On any ward, there are usually multiple staff who also change every shift. There will be colour-coded clothing, but, even so, at times I got confused with the unform coding system. Every time a staff member sees a patient, they should give their name (first or second name would suffice) and their role. If the patient is in a single room with a whiteboard, the coding system could be displayed on that whiteboard. My ward had 32 beds, 16 as single rooms, and four bays of four beds each. Names of patients were not on the doors of the single rooms, nor outside the bays. Unless there is a good reason otherwise, such names should be there. Each nurses’ station had a large screen display of patients’ names, named consultant, and named nurses. It should be easy to add a photo of the patient to the screen. It would also be useful to have a column which highlighted any potential patient safety risks, e.g. ‘Misidentification Risk’ if there were two patients with the same name on the ward, or the name was particularly common and might be confused with a patient on another ward (e.g. Smith).

Patients will usually have a named consultant and named nurses specifically looking after them, as well as other members of the clinical team. If there is a TV video system by the patient’s bed, it should be possible to create software that will automatically display on the screen staff who are on the ward at present, along with highlighted named staff allocated to the patient.

Single rooms, as well as beds in bays, will now usually have technical items, ranging from a call handle with several buttons, TV monitor, etc. These should be explained in person with the individual, with appropriate leaflet(s) nearby.

My wrist name band had my **surname** in bold, and my date of birth as dd/mm/yyyy. There is a simple psychology to labelling, some of which has been summarised in formal reports [3]. My view is that the **SURNAME** should be in uppercase bold, and that the date of birth should be dd/month/yyyy so as to avoid any confusion with the American system, whereby September 11 is 11/09 in the UK but 9/11 in USA. If that is not possible, then at least have the month **number** in bold font. All of this will help prevent misidentification disasters.

Performance data for individual UK cardiac surgeons is now readily available for patients to see. Should this be more detailed in respect of specific procedures, and should it apply to all surgical specialities? I simply pose that as a question.

Many staff in hospitals are now foreign-trained, and some have distinctive accents which at times may be difficult to follow. With COVID-related mask mandates still in operation, and likely to be for months or years to come, the issue arises as to how well patients may understand what they are being told. According to Professor Neil Ferguson at Imperial College (BBC interview, July 18, 2021), the value of masks is primarily to prevent virus transmission, and even for that purpose they are only around 20% effective; they may be minimally effective for preventing infection by the virus – e.g. there is some evidence that eyes may also be a source of virus entry [4]. There would therefore seem to be good grounds for doing a simple experiment in a hospital or simulated environment – one ward where staff wear masks as at present, one ward where they do not wear masks, and one ward where they wear clear masks and / or a face shield. The second and third options, if proved equally preventative, would allow for better communication between patients and staff, as well as alleviating the discomfort of wearing a mask.

During my hospital stay, I found that the psychology of environmental design [5] could be improved. Naturally, this may relate to architecture, building and equipment manufacturer professions, but someone on the hospital staff should be trained in such matters. As a simple example, the door to my single room and to the adjacent bathroom had identical features on both sides. Thus, I did not know whether to push or pull the door handle. Having PUSH/PULL signs would have made that clear.

At discharge, many hospitals have some form of feedback system. Ideally, a Feedback Interview should be conducted in person using a format which covers areas such as staff, ease of use of amenities, food/drink, etc. Ways in which gratitude can be expressed or further feedback given could also be provided. When a patient is discharged, they should be given the mobile number of a key member of staff (e.g. house officer on the ward) whom they could contact for up to 4 weeks after discharge if they had a particular query. Queries later than this might reasonably be expected to be handled by the GP.

Finally, one of the papers emanating from my work with CORESS compared patient safety with aviation safety [1]. Cockpit voice recorders have been standard in the airline industry for many decades. With the advent of research on using head-mounted video recording systems such as GoPro during surgery [6], will we get to the stage where patients will ask for a video recording of their operation which they can then take home with them!

## Conclusions

Human factors lessons for patient safety can be learned by drawing on the experience of patients who undergo surgery, especially those patients who have relevant knowledge, skills and experience. Even simple changes to the design of hospital environments can make a significant difference to the safety and wellbeing of patients in hospital.

## Data Availability

N/A

## Abbreviations

GP: General Practitioner
CORESS: Confidential Reporting System for Surgery
